# Assessing the Influence of Guanidinoacetic Acid on Growth Performance, Body Temperature, Blood Metabolites, and Intestinal Morphometry in Broilers: A Comparative Sex-Based Experiment

**DOI:** 10.3390/ani14131853

**Published:** 2024-06-22

**Authors:** Abdulaziz A. Al-Abdullatif, Mahmoud M. Azzam, Emad M. Samara, Mohammed A. Al-Badwi, Xinyang Dong, Abdel-Moneim Eid Abdel-Moneim

**Affiliations:** 1 Animal Production Department, College of Food and Agriculture Sciences, King Saud University, Riyadh 11451, Saudi Arabia; aalabdullatif@ksu.edu.sa (A.A.A.-A.); dremas@ksu.edu.sa (E.M.S.); malbadwi@ksu.edu.sa (M.A.A.-B.); 2 Animal Science College, Zhejiang University, Zijingang Campus, Hangzhou 310058, China; sophiedxy@zju.edu.cn; 3 Biological Applications Department, Nuclear Research Center, Egyptian Atomic Energy Authority, Abu-Zaabal 13759, Egypt; aeabdelmoneim@gmail.com

**Keywords:** guanidinoacetic acid, growth, body temperature, sex, broilers

## Abstract

**Simple Summary:**

This experiment investigated how male and female broiler chickens respond to a dietary guanidinoacetic acid (GAA) supplementation concerning their growth, body temperature, blood components, carcass traits, and intestinal structure. The results indicated that male broilers grew faster and consumed feed more than females. When given a higher dose of GAA, all broilers gained weight better and had improved feed efficiency. Further, GAA improved intestinal architecture, especially in male broilers. Interestingly, male birds treated with GAA had higher shank and head temperatures compared to females. Overall, this research suggests that GAA supplementation, especially at a higher dose, can enhance broiler growth and intestinal health, which could be beneficial for broilers.

**Abstract:**

It is well known that female and male broilers showcase variations in their growth performance, influenced by various physiological factors. This experiment aims to explore potential differences between female and male broilers concerning growth performance, body temperature, blood metabolites, carcass traits, and intestinal architecture in response to guanidinoacetic acid (GAA) supplementation. A total of 240 Ross 308 broiler chickens were arranged in a 3 × 2 factorial design and randomly allocated into 48 boxes, each containing 5 birds. The experiment comprised six treatments, with eight replicates per treatment. The main factors investigated were dietary GAA levels (0%, 0.06%, and 0.12%) and sex (male and female). Male broilers demonstrated superior body weight gain (BWG) and feed intake (FI) compared to females (*p*< 0.05). GAA supplementation at 0.12% concentration notably improved BWG and reduced FI and feed conversion ratio (FCR) across experimental phases (*p* < 0.05). However, interactions between sex and GAA were minimal except for reduced FI and FCR (*p* < 0.05) in both sexes during early growth stages. Regardless of GAA treatment, the male birds exhibited more elevated shank and head temperatures than the females. Carcass traits were largely unaffected by GAA supplementation or sex, except for higher heart yield in the males. Serum metabolite levels were not different between treatment groups at 10 and 24 days of age, except for a higher level of serum creatinine at 10 days in the female birds with 0.06% GA supplementation (*p* < 0.05). Intestinal morphology was significantly affected by GAA and sex, depending on the segment of intestine, in which GAA supplementation significantly increased villus height, crypt depth, villus width, surface area, and goblet cell count, while the males consistently exhibited higher values of these parameters than the females, and differences were observed between intestinal segments, especially in the ileum and duodenum, at different ages. In conclusion, the interactions between GAA and sex had minimal influences on growth performance indices. However, male broilers demonstrated a more pronounced response to GAA concerning ileal architecture. This study highlights the importance of supplementing broiler chicken diets with GAA for optimizing male broiler performance and intestinal function. The inclusion of GAA into broiler diets needs further study to reveal the underlying mechanisms driving these sex-specific responses and assess the long-term impacts of GAA supplementation on broiler health and productivity.

## 1. Introduction

Poultry production plays a critical role in meeting the escalating demand for protein consumption worldwide. With the global population projected to surpass 9.7 billion by 2050, efficient and sustainable animal farming practices are essential for ensuring food security and addressing nutritional needs [1]. Poultry meat production has experienced substantial growth over the past five decades, becoming a dominant player in the global meat industry, and currently, it contributes to approximately one-third of the total meat production worldwide [2]. Projections indicate that the poultry sector will continue to drive the expansion of meat production in the upcoming decade, and it is expected to contribute to half of all additional meat produced during that period [3]. In 2020, poultry meat output surged to 137 million tons, marking a notable 2.6% increase compared to the previous year [4]. Broiler chickens, in particular, are valued for their rapid growth and high feed conversion efficiency, making them a primary protein source for human consumption. As such, optimizing broiler performance and health through targeted nutritional interventions is paramount to meeting the growing demands of the poultry industry [5].

Nutritional intervention strategies, including early feeding and dietary supplementation, have emerged as key approaches to enhance broiler production efficiency and profitability [6,7]. Guanidinoacetic acid (GAA), a precursor of creatine (CREAT), has garnered attention for its potential benefits in improving growth performance, muscle development, and overall health in broiler chickens [8]. By supporting energy metabolism and protein synthesis, GAA supplementation offers promising prospects for maximizing broiler productivity and carcass quality while minimizing production costs [9]. In fact, GAA is vital for energy metabolism, particularly in muscle cells, through the CREAT and phosphocreatine (PCr) system, which stores and releases energy as needed. About 1.7% of the CREAT and PCr pool converts to creatinine daily, necessitating continual replenishment [10]. CREAT can be obtained from dietary animal protein or synthesized endogenously. The synthesis involves combining L-arginine with glycine to produce L-ornithine and GAA in the pancreas and kidneys, then methylation of GAA by S-adenosyl-methionine (SAM) generates CREAT, transported to tissues by a sodium-linked transporter. Poultry feeding non-animal protein diets may face CREAT deficiency, making supplementation with CREAT or GAA crucial. GAA, requiring only a methyl-group transfer from SAM, may conserve dietary L-arginine and benefit rapidly growing chickens by facilitating CREAT supply to muscles [11]. ATP regeneration from the CREAT and PCr systems is vital in cardiac energy management [12]. While CREAT is used as a feed additive, challenges like instability and cost favor GAA [13]. However, GAA methylation to CREAT increases SAM demand, potentially leading to homocysteine accumulation or deficiencies in methionine, vitamin B12, folic acid, or choline [14].

Male and female broilers often exhibit differences in growth performance and physiological responses to dietary interventions. These sex-related variations underscore the importance of considering sex-specific nutritional strategies to optimize broiler performance and intestinal function in commercial poultry farming [15]. Understanding the unique metabolic and physiological characteristics of male and female broilers is crucial for designing targeted nutritional interventions that can effectively address sex-specific requirements and enhance overall productivity in broiler production systems. Nevertheless, despite the growing interest in GAA supplementation as a nutritional strategy for broiler production, limited research has explored its effects on male and female broilers’ growth performance, carcass traits, body temperature, blood metabolites, and intestinal morphometry. Therefore, the present study aims to investigate the comparative influences of dietary GAA supplementation on these parameters in male and female broilers.

## 2. Materials and Methods

### 2.1. Chickens, Diets, and Experimental Design

A total of 240 day-old Ross 308 broiler chickens were procured from the hatchery of Al Wadi Poultry Company, in Riyadh, Saudi Arabia. Upon arrival, the chickens underwent feather sexing, and weighing, and were organized into 3 × 2 factorial arrangements. Subsequently, they were randomly allocated into 48 boxes (58 cm length × 50 cm width × 35 cm height), each accommodating 5 birds. The experiment comprised 6 treatments, each replicated 8 times, investigating dietary levels of GAA (0%, 0.06%, and 0.12%) and the sex of the broilers (male and female). GAA was introduced by incorporating the feed additive CreAMINO (>96% GAA; Alzchem Group, Chemiepark, Trostberg, Germany) on top of the feed. Throughout the experiment, birds had ad libitum access to water and feed, supplied as a starter (1–10 d) and grower (11–24 d) mash corn–soybean meal basal diets (Table 1). To maintain uniform conditions for all birds, pens were situated within the same controlled environment shed, equipped with nipple drinkers and feeders. Environmental parameters such as lighting, ambient temperature, and relative humidity were maintained according to the guidelines of Ross 308. Experimental diets were analyzed using [16] as described by [17,18] to determine the proximate analysis of experimental diets and total lysine, methionine, cysteine, and threonine

### 2.2. Growth Performance

Body weight and feed intake (FI) were assessed on days 1, 5, 10, and 24 on a per-pen basis. Body weight gain (BWG) and feed conversion ratio (FCR) were then computed as feed intake divided by the BWG (g/g) for the experimental phases.

### 2.3. Thermophysiological Measurements

On days 5, 10, and 15 of age, two chickens per replicate were randomly chosen for thermophysiological measurements as described by Abudabos et al. [19]. In brief, twice a day (at 08:00 and 14:00 h), the cloacal temperature was measured using a digital thermometer with a short probe (Geratherm Medical, Geratal, AG Germany, GT-131) calibrated to the nearest 0.1 °C. Meanwhile, infrared thermographic images (thermograms) of the left side of the head, surface body, and shank were obtained using a forward-looking infrared camera (Traceable Mini IR™ Thermometer, Friendswood, TX, USA), positioned approximately 50 cm from the chick’s surface. These thermograms were then analyzed using specialized software (TherMonitor, Thermoteknix Systems Ltd., Cambridge, UK), employing a rainbow color scheme for visualization.

### 2.4. Carcass Traits

At the age of 24 days, eight birds per group were randomly chosen for carcass assessment and sampling. Following a 12 h fasting period, the birds were slaughtered by clean cut to the jugular vein, carotid artery, and windpipe. Subsequently, organs including the breast, leg, heart, liver, kidney, pancreas, gizzard, spleen, proventriculus, bursa of Fabricius, and thymus were individually separated and weighed to determine their relative weight to the live body weight.

### 2.5. Blood Biochemical Measurements

Blood samples were obtained from the birds’ wing veins at 10 and 24 days old for biochemical analysis. These samples underwent centrifugation at 1500× *g* for 20 min and 5 °C to separate the sera, which were then stored at −80 °C. Hormones (triiodothyronine and thyroxine) were measured through analyzed serum total triiodothyronine(T3) and thyroxine (T4) hormones using ELISA Kit (CTK Biotech, San Diego, CA, USA).Quantitative assessment of serum concentrations of total protein, globulin, albumin, glucose, cholesterol, uric acid, and creatinine was conducted using colorimetric methods following the manufacturer’s protocols from the Randox kits (RANDOX Laboratories Ltd., Crumlin, UK) using a microplate reader (MR-96A; Mindray Bio-Medical Electronics Co., Ltd., Shenzhen, China).

### 2.6. Histomorphometric Evaluation

At 10 and 24 days of age, samples from the duodenum, jejunum, and ileum were gathered, rinsed with 0.9% saline solution, and immersed in a 10% formalin solution for fixation. Tissues were embedded in paraffin, and 20 cross-sections 4 μm thick of each sample were sliced. Following the Bancroft and Gamble [20] protocol, the sections were stained with hematoxylin and eosin. The stained tissues were examined under a light microscope (Nikon microscope, Nikon Corp, Tokyo, Japan), and representative fields were photographed (Olympus digital video camera, DP72, Aartselaar, Belgium) for morphometric analysis using CellSens software, version 1.16. Ten well-oriented villi in each sample were selected to measure villus height (VH), crypt depth (CD), villus width, and goblet cell count. The VH/CD ratio and villus surface area were subsequently calculated as described by [21]. Goblet cells were counted per villi from base towards the tip of the villi visually under the microscope using 200× magnifications.

### 2.7. Statistical Analysis

The gathered data underwent normality testing using the Shapiro–Wilk test using SPSS software (version 19.0; SPSS Inc., Chicago, IL, USA). The normally distributed data were then analyzed using the General Linear Model procedure of SPSS, following a 3 × 2 factorial arrangement of treatments, to evaluate the main impacts of GAA level and sex and their interactions. For significance identifications among means, Tukey’s multiple comparison test was employed at *p* < 0.05. Furthermore, intestinal histomorphometry data were subjected to three-way ANOVA to determine the influence of different intestinal segments and their interaction with the aforementioned main factors.

## 3. Results

### 3.1. Growth Performance

Data in Table 2 illustrate the influence of sex and GAA supplementation on the growth performance of broiler chickens. Our findings indicate that supplementation with GAA at 0.12% concentration resulted in improved weight gain during the 11–24 day of age as well as the overall one (*p* < 0.05). Additionally, FI and FCR were notably reduced in the GAA-treated groups compared to the control across the experimental phases (*p* < 0.05). Interestingly, while there was no significant interaction observed between sex and GAA supplementation in most studied parameters, there was a notable exception concerning FI and FCR at 6–10 days (*p* < 0.05). In this instance, both male and female birds in the GAA-treated groups exhibited reduced FI and FCR compared to the control group. Furthermore, the male broilers exhibited superior weight gain and feed intake throughout the experimental periods compared to the females.

### 3.2. Thermophysiological Measurements

**Figure 1:**
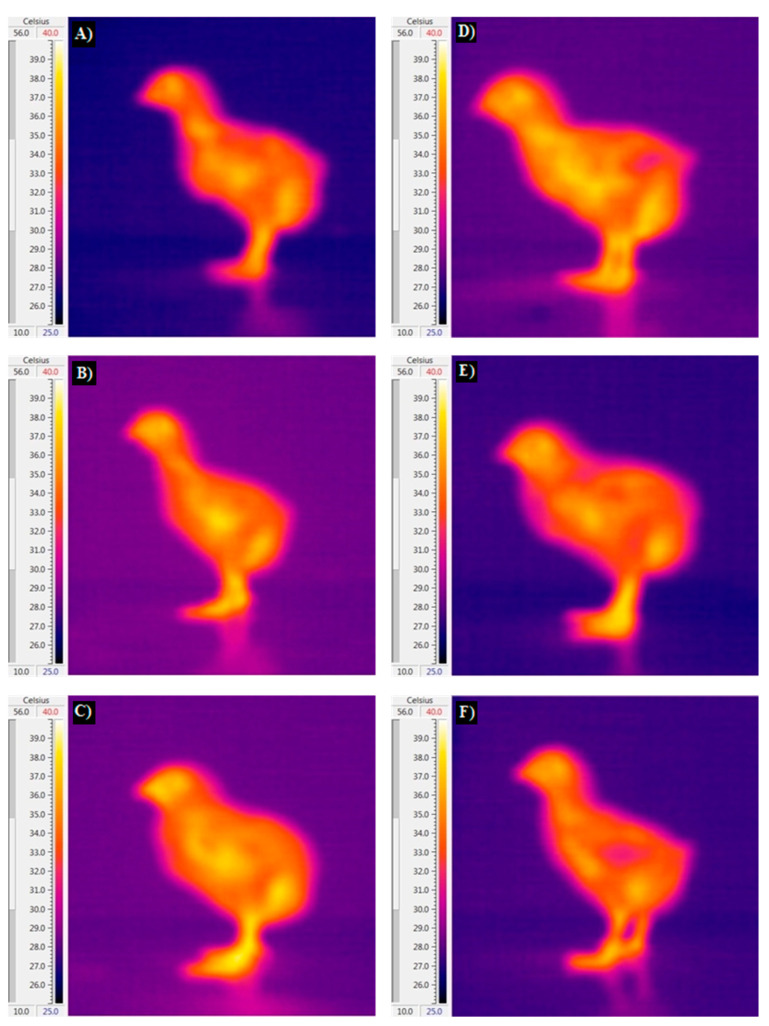
Impact of guanidinoacetic acid on body part temperatures of male (the right) and female (the left) broiler chickens at 5 days of age. Experimental groups: control group (**A**,**D**), 0.06% GAA (**B**,**E**), and 0.12% GAA (**C**,**F**).

**Figure 2:**
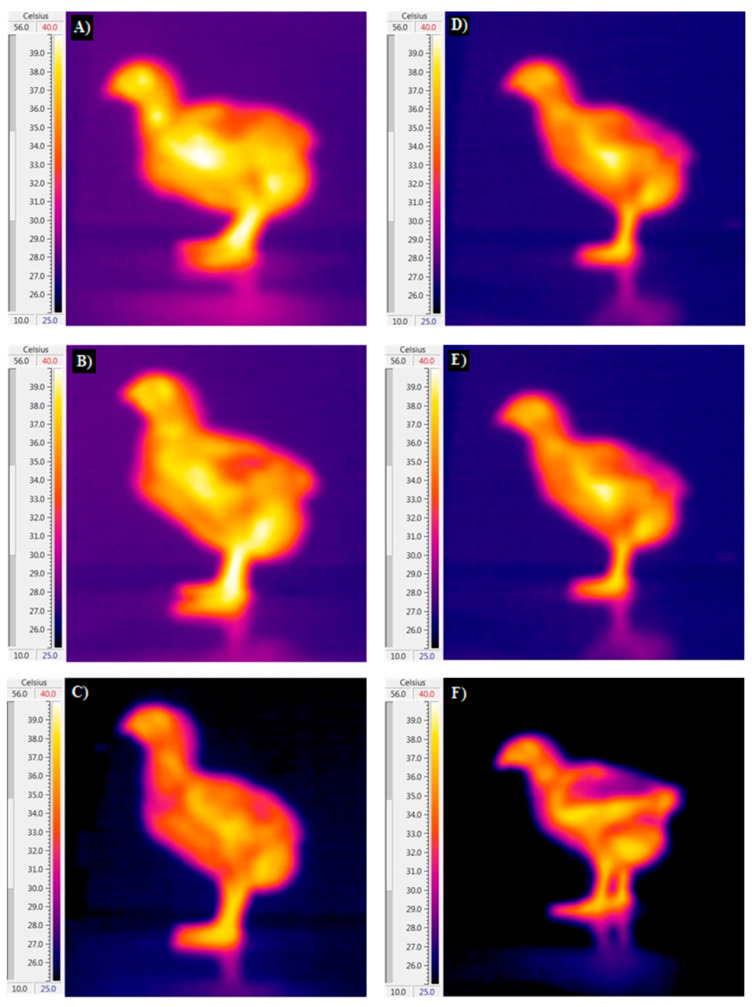
Impact of guanidinoacetic acid (GAA) on body part temperatures of male (the right) and female (the left) broiler chickens at 10 days of age. Experimental groups: control group (**A**,**D**), 0.06% GAA (**B**,**E**), and 0.12% GAA (**C**,**F**).

**Figure 3:**
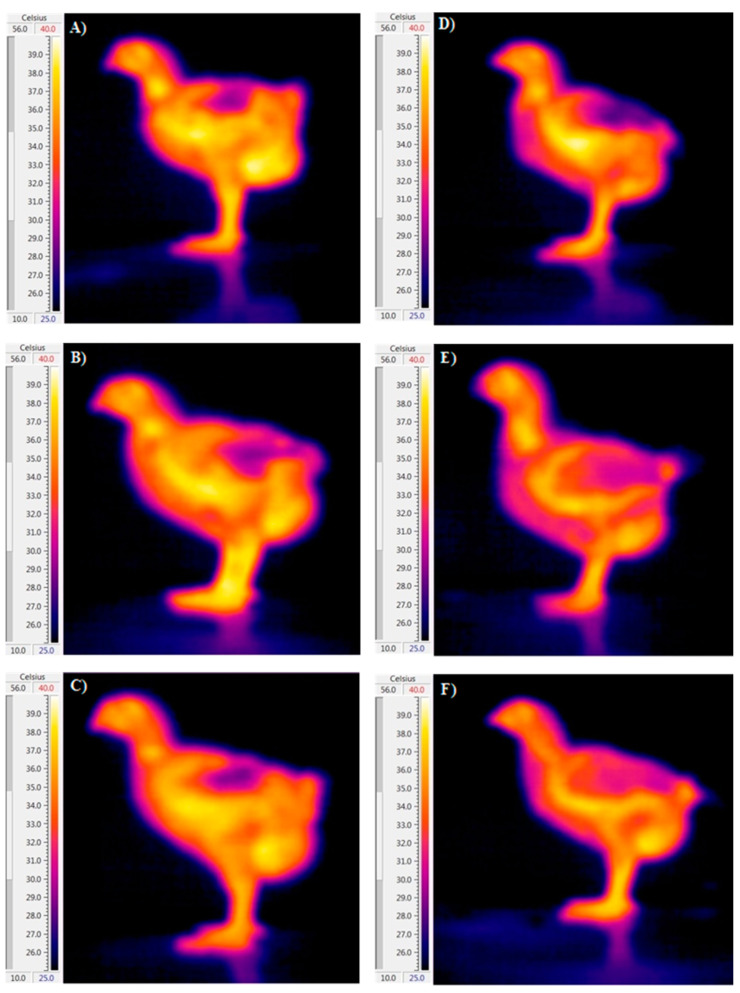
Impactof dietary guanidinoacetic acid (GAA) on body part temperatures of male (the right) and female (the left) broiler chickens at 15 days of age. Experimental groups: control group (**A**,**D**), 0.06% GAA (**B**,**E**), and 0.12% GAA (**C**,**F**).

The influence of dietary inclusion of GAA, sex, and their interaction on body temperature, including cloaca, surface, head, and shank temperatures, are presented in Table 3 and Figure 1, Figure 2 and Figure 3. Our analysis indicates that neither GAA supplementation nor the interaction between GAA and sex significantly influenced the temperatures of the body parts examined. However, we observed notable differences in shank and head temperatures between male and female birds at specific time points (*p* < 0.05). Specifically, male birds exhibited elevated shank temperatures at 10 and 15 days, and elevated head temperatures at 15 days, compared to their female counterparts.

### 3.3. Carcass Traits

As illustrated in Table 4, the analysis of carcass traits reveals that neither GAA supplementation, sex, nor their interaction significantly influenced most of the measured parameters. However, one notable exception was observed in the heart weight (%), which was found to be higher in male broilers compared to females.

### 3.4. Serum Metabolites

The influences of dietary inclusion of GAA, sex, and their interaction on serum metabolites at 10 and 24 days of age are summarized in Table 5 and Table 6. Our results indicate that serum levels of total protein, globulin, albumin, glucose, uric acid, cholesterol, and T3 and T4 hormones were not influenced by GAA supplementation, sex, or their interaction at either age. Female birds fed diets supplemented with 0.06% GAA recorded a higher level of serum creatinine at 10 days (*p* = 0.003) with no alteration at 24 days of age among the groups.

### 3.5. Intestinal Histomorphometry

The influences of dietary inclusion of GAA, sex, intestinal segment, and their interaction on intestinal morphometry parameters at 10 and 24 days of age are shown in Table 7 and Table 8. The results revealed significant main effects for GAA supplementation, sex, and intestinal segment, indicating that all three factors independently influence histomorphometry parameters, including VH, VW, CD, VH/CD ratio, SA, and GC.

GAA supplementation led to significant increases in all the aforementioned parameters compared to the control group at both ages except CD on day 10 and VH/CD on day 24. Similarly, male broilers exhibited higher values of these measures compared to their female counterparts at both ages. Regarding intestinal segments, the duodenum recorded higher VH values, while the ileum recorded higher VW, CD, SA, and GC compared to other intestinal parts at 10 days. However, at 24 days, higher VH, VW, CD, and SA were noticed in the ileum.

Interactions between factors further elucidated the complexity of the relationships. At 10 days of age, the interaction between GAA and sex was noticed in VH and VW, with the highest VH values recorded in the GAA-treated male birds and for VW in the female birds treated with 0.06% GAA. Similarly, the interaction between GAA and the gut segment was observed in all the parameters except for CD and SA, with the highest VH noticed in the duodenum of treated birds. The triadic interaction was noticed in all parameters except for VH and GC.

At 24 days of age, the interactions between GAA and sex, and between GAA and intestinal segment, were observed in all parameters except for VW and SA, while the interaction between sex and gut segment was observed in VH, CD, and GC. The triadic interaction was noticed in all parameters except for the VH/CD ratio. The highest VH values were recorded in the ileum of the treated male birds.

## 4. Discussion

The growing cycle for chickens in Saudi Arabia varies from 21 to 28 days, with the average live weight ranging from approximately 1200 to 1600 g and the average carcasses weight for chickens ranging from approximately 700 to 1200 g. Broiler chickens, being fast-growing animals, necessitate higher CREAT levels compared to adult birds due to their elevated requirements for muscle development, tissue growth, ATP regeneration, and compensating for CREAT losses to creatinine [22]. While carnivorous or omnivorous animals typically acquire around half of their CREAT needs from their diet, with the remainder being synthesized internally [23], broiler chickens in modern commercial production, because of being herbivores, do not derive CREAT from their diets. Consequently, their capacity for “de novo” synthesis may be constraining, particularly as highly productive animals. Recent research findings indicate that the supplementation of broiler diets with varying levels of GAA led to a range of influences on growth performance, transitioning from enhanced growth to negligible impacts [24,25,26].

The results of the present experiment reveal that supplementing GAA improved BWG and reduced FI and FCR but did not affect carcass characteristics. Furthermore, male broilers exhibited superior BWG and FI throughout the experimental periods compared to females. However, no interaction was observed between sex and GAA supplementation in growth performance and carcass traits. Our results are consistent with those of Cao et al. [27] who reported that dietary supplementation with GAA did not affect BW, BWG, or carcass traits and enhanced FCR, particularly in low-metabolizable-energy diets. Additionally, Mousavi et al. [28] observed that supplementing GAA improved FCR without noticeably affecting BW in broiler-fed diets with varying energy content. The findings of De Souza et al. [28] also suggest that GAA supplementation during the starter phase can enhance broiler chicken growth performance. Heger et al. [29] concluded that the influence of GAA supplementation on BW or BWG was minimal while emphasizing that FCR proved to be a more sensitive indicator, exhibiting a positive response to GAA in broiler diets. Conversely, both Khajali et al. [30] and Ceylan et al. [25] reported a negligible impact of GAA supplementation on growth performance or carcass traits in broilers.

The enhancement in growth performance observed with GAA inclusion may be attributed to its ability to conserve arginine and glycine, redirecting these amino acids toward functions such as protein synthesis [8,31,32,33], while also negatively modulating endogenous GAA formation [34]. Both DeGroot et al. [32] and Yazdi et al. [35] demonstrated that dietary GAA increases CREAT concentration, which is advantageous and cost effective over direct supplementation of CREAT due to the inhibitory influence of high dietary CREAT on the activity of L-arginine: glycine amidinotransferase enzyme via negative feedback. However, the activity of guanidinoacetate N-methyltransferase remains unaffected by CREAT, allowing GAA to serve as a precursor for CREAT biosynthesis in the liver, particularly benefiting fast-growing broiler chickens with heightened CREAT demands for muscle energy maintenance [8,12]. Furthermore, dietary GAA increases ATP, PCr concentrations, and the PCr/ATP ratio [32,35], thereby potentially enhancing various energy-dependent processes such as ionic homeostasis, cellular motility, and muscle contractions [36]. Considering that male broilers typically exhibit higher energy requirements than females due to their faster growth rate and greater muscle mass development [37], the improvement in the energetic status of male broiler muscle could markedly contribute to their superior growth compared to females in this experiment.

Neonatal broiler chicks are particularly sensitive to thermoregulation early in life due to limited physiological mechanisms for maintaining body temperature, including underdeveloped thermoregulatory systems and reliance on external heat sources [38]. Their high metabolic rate and narrow critical temperature range increase susceptibility to heat loss or stress, impacting growth, immune function, and mortality rates [39]. We hypothesized that GAA, by influencing metabolism and energy utilization, could enhance thermoregulation responses in neonatal broiler chicks. As a precursor to CREAT, GAA facilitates rapid ATP regeneration during high-intensity activities like shivering thermogenesis, improving energy production and utilization in thermoregulatory processes [12]. GAA supplementation may also increase heat production by improving CREAT availability, aiding in maintaining body temperature in varied environments [10]. Therefore, to the best of our knowledge, for the first time, we investigated the impact of GAA on the thermophysiological conditions of neonatal broiler chicks. Nevertheless, our results revealed that GAA did not substantiallyaffect the cloacal and body part temperatures. However, notable elevations in the shank and head temperatures were observed in male chicks compared to females. This could be attributed to their larger body size, breast muscle development, and higher metabolic rate [40,41], indicating increased thermoregulatory needs as they grow. The increase in the surface area available for heat exchange with the increase in birds’ body mass might be another explanation [42]. However, the lack of noteworthy GAA supplementation in this experiment may be due to the chickens being raised in a thermoneutral environment and without experiencing heat or cold stress, or the doses of GAA examined were not suitable to exert a notable impact. Further research is needed to investigate GAA’s role in enhancing the thermoregulation of broilers under stress conditions, offering potential benefits for poultry production welfare.

GAA supplemented in broiler diets is absorbed in the intestine and transported to the liver via the bloodstream [33], where it is converted into CREAT and then into creatinine, a non-nutritive waste product excreted by the kidneys [34]. Based on the findings of the present experiment, we found irrelevant changes in serum creatinine and uric acid levels with GAA supplementation at 24 days of age, consistent with findings by Khalil et al. [43] and Wyss and Kaddurah-Daouk [10]. Similarly, He et al. [44] observed no alteration in serum creatinine concentrations in pigs fed diets supplemented with GAA. Nevertheless, Cao et al. [27] reported decreased serum creatinine levels with GAA supplementation at 0.08%, attributing it to enhanced energy metabolism in tissues that required a high energy requirement. Furthermore, administering GAA did not adversely affect hepatic function biomarkers or thyroid hormone concentrations, indicating no harmful influences on liver and thyroid activity. Gao et al. [45] and Stoll et al. [46] reported that serum albumin concentration is a potent indication of hepatic protein metabolic status, protein anabolism, and the nutritional condition in broilers. This observation correlated with enhanced growth performance noticed in this experiment. These findings align with studies by Cao et al. [27], who found no alterations in hepatic enzymes in the birds fed diets with GAA ranging from 0.02% to 0.08% compared to those that were fed normal and low-metabolizable-energy diets. Similarly, Khalil et al. [43] and Amiri et al. [47] reported meaningless alterations in thyroid hormone activities with GAA inclusion.

In the current experiment, the dietary inclusion of GAA at concentrations of 0.06% and 0.12% led to enhanced intestinal architecture across various segments in both male and female broilers. The interaction observed between sex, GAA supplementation, and intestinal segment highlights the intricate relationship among these factors in shaping the intestinal health in broilers, underscoring the importance of considering multiple variables for optimizing gut function in poultry production. While previous research on the influencesof GAA supplementation on intestinal morphology in poultry under thermoneutral conditions is limited, some studies have shown crucial improvements. For instance, Emami et al. [48] demonstrated that GAA supplementation at 1.2 g/kg increased the SA of jejunal villi in birds under cold temperature conditions. Similarly, Amiri et al. [47] observed improvements in VH, VW, and SA in both the duodenum and jejunum of broilers fed GAA at 0.06% and 0.12%. Ahmadipour et al. [49] also reported increased VH, VW, and SA in various parts of the small intestine with GAA inclusion at 0.1 to 0.2%. Despite these findings, the precise mechanism by which GAA enhances intestinal morphology remains unclear. However, since GAA is synthesized from arginine, its positive impacts may be attributed to arginine’s beneficial impacts on intestinal health. Arginine has been shown to upregulate gene expression in the target of the rapamycin signaling pathway, stimulating protein synthesis and reducing protein degradation in chicken intestinal epithelial cells [50]. Additionally, arginine exhibits anti-inflammatory activity [51] and promotes intestinal innate immunity while maintaining the homeostasis of the intestinal microbiota [52]. This may contribute to an increase in goblet cell count, thereby improving barrier function and mucin production throughout the gut. Khajali et al. [53] also noted positive influences of arginine supplementation on the VH and VH/CD ratio in the small intestine of broilers. In fact, the improvement in intestinal morphology may explain the enhanced growth performance observed in broilers fed GAA-supplemented diets in the present experiment.

## 5. Conclusions

While male broilers generally exhibited superior BWG and FI compared to females, GAA supplementation, particularly at 0.12%, notably improved BWG and reduced FI and FCR across experimental phases. Future research is needed to reveal the underlying mechanisms driving these sex-specific responses and assess the long-term impacts of GAA supplementation on broiler health and productivity.

## Figures and Tables

**Table 1:** Ingredients and nutrient levels of the basal diet at different growth phases.

Ingredients, %	Starter (d 1–10)	Grower (d 11–24)
Corn	528.60	575.80
Soybean meal CF, 48%	391	339.8
Plant oil	37.2	44.10
Dicalcium phosphate.	18.2	16.30
Limestone	10.0	9.30
Salt	4.20	3.20
DL-Methionine	3.50	3.20
L-Lysine HCL	2.0	1.90
L-Threonine	1.30	1.10
Choline Cl 60%	0.90	0.90
Sodium bicarbonate	0.10	1.40
Premix Blank	3.00	3.00
Total	1000	1000
Calculated values ^1^, %		
Crude protein	23.29	21.15
Crude fat	6.51	7.26
Crude fiber	2.83	2.72
Calcium	0.96	0.87
Phosphorus	0.73	0.67
Available phosphorus	0.48	0.44
Potassium	1.05	0.95
Sodium	0.16	0.16
Chloride	0.28	0.22
Dig. lysine	1.28	1.15
Dig. methionine and cysteine	0.95	0.87
Metabolizable energy, kcal/kg	3130	3178
Analyzed values, %		
Crude protein	23.70	21.30
Crude fiber	3.30	3.27
Total Lysine	1.50	1.34
Total methionine and cysteine	1.04	0.93
Total threonine	1.02	0.92

Premix Per 1 Kg: thiamine (B1), 2 mg; riboflavin (B2), 6 mg; niacin (B3), 50 mg; pantothenic acid (B5), 15 mg; pyridoxine (B6), 3 mg; biotin (B7), 150 µg; folic acid, 1.75 mg; cobalamins (B12),16.0 µg; K3 (MNB), 3 mg; D3 (cholecalciferol), 5000 IU; A (retinol acetate), 10,000 IU; E (Dl-alpha-tocopherol acetate), 50 IU; total antioxidants, 50;manganese (oxide), 120; zinc (oxide), 100; iron (sulfate), 40; copper (sulfate), 16; iodine (potassium iodide), 1.25; selenium (sodium selenite) 0.30. ^1^ Values were calculated from data provided by the BESTMIX^®^ (adifo software, 2020, Maldegem, Belgium).

**Table 2:** Impact of guanidinoacetic acid (GAA) on growth performance of male and female broiler chickens.

GAA, %	Sex	IBW, g	WG, g/Bird/Period				FI, g/Bird/Period				FCR, g Feed/g Gain			
			Days 0–5	Days 6–10	Days 11–24	Days 0–24	Days 0–5	Days 6–10	Days 11–24	Days 0–24	Days 0–5	Days 6–10	Days 11–24	Days 0–24
0	M	46.69	81.90	172.4	1021.6	1275.9	70.79	199.2 ^a^	1323.2	1593.1	0.865	1.157 ^a^	1.295	1.249
	F	46.63	75.65	166.4	882.1	1124.2	67.27	180.9 ^b^	1175.3	1423.5	0.891	1.088 ^b^	1.333	1.267
0.06	M	46.58	82.52	176.1	1017.1	1275.7	71.13	186.6 ^b^	1291.3	1549.0	0.863	1.060 ^b^	1.270	1.214
	F	46.68	78.39	170.3	900.6	1149.3	70.75	183.8 ^b^	1138.8	1393.3	0.905	1.080 ^b^	1.266	1.213
0.12	M	46.62	80.65	172.1	1035.6	1288.4	69.69	184.1 ^b^	1286.8	1540.6	0.865	1.070 ^b^	1.244	1.196
	F	46.59	75.85	171.1	915.7	1162.6	67.44	179.5 ^b^	1132.7	1379.7	0.892	1.051 ^b^	1.237	1.187
SEM		0.045	0.714	1.175	9.976	10.50	0.507	1.466	18.75	13.58	0.007	0.009	0.017	0.007
Sex	M	46.63	81.69 ^a^	173.5 ^a^	1024.8 ^a^	1280.0 ^a^	70.54 ^a^	189.9 ^a^	1300.4 ^a^	1560.9 ^a^	0.864 ^b^	1.095	1.270	1.220
	F	46.63	76.63 ^b^	169.3 ^b^	899.5 ^b^	1145.4 ^b^	68.49 ^b^	181.4 ^b^	1148.9 ^b^	1398.8 ^b^	0.896 ^a^	1.073	1.279	1.222
SEM		0.067	0.886	1.313	5.463	4.919	0.675	1.685	8.529	8.921	0.010	0.010	0.011	0.008
*p-*value														
GAA		0.90	0.33	0.26	0.04	0.01	0.11	0.02	0.02	0.009	0.92	0.002	0.001	>0.001
Sex		0.95	>0.001	0.02	>0.001	>0.001	0.03	0.001	>0.001	>0.001	0.03	0.11	0.56	0.84
GAA × Sex		0.74	0.78	0.47	0.43	0.23	0.41	0.02	0.97	0.90	0.87	0.043	0.42	0.62

Means followed by different letters in the same column are significantly different (*p* < 0.05). SEM = standard error of means, IBW = initial body weight, WG = weight gain, FI = feed intake, FCR = feed conversion ratio, M = male, F = female.

**Table 3:** Impact of guanidinoacetic acid (GAA) on body temperature (°C) of male and female broiler chickens.

GAA, %	Sex	Cloacal, °C			Body Surface, °C			Head Surface, °C			Shank Surface, °C		
		Day 5	Day 10	Day 15	Day 5	Day 10	Day 15	Day 5	Day 10	Day 15	Day 5	Day 10	Day 15
0	M	40.50	40.51	40.86	37.05	38.93	38.50	36.42	36.35	36.93	36.50	38.38	37.83
	F	40.46	40.63	40.98	37.23	38.09	38.36	36.53	36.36	36.23	36.98	37.54	37.11
0.06	M	40.49	40.55	40.90	37.03	38.66	38.65	36.58	37.10	36.61	37.29	37.85	37.16
	F	40.59	40.66	41.06	37.03	37.93	38.18	36.53	36.35	36.58	37.50	37.23	37.26
0.12	M	40.84	40.53	40.94	36.98	38.53	38.64	37.09	37.10	36.51	37.21	38.08	37.46
	F	40.71	40.48	40.93	36.85	37.73	38.35	36.15	36.35	36.28	37.20	37.11	36.89
SEM		0.050	0.034	0.024	0.163	0.145	0.091	0.134	0.214	0.079	0.153	0.132	0.088
Sex	M	40.61	40.53	40.90	37.02	38.70	38.60	36.70	36.82	36.68 ^a^	37.00	38.10 ^a^	37.48 ^a^
	F	40.59	40.59	40.99	37.03	38.04	38.30	36.40	36.30	36.24 ^b^	37.23	37.29 ^b^	37.09 ^b^
SEM		0.068	0.049	0.034	0.242	0.192	0.131	0.191	0.311	0.104	0.218	0.171	0.114
*p*-value													
GAA		0.040	0.452	0.532	0.869	0.776	0.932	0.909	0.787	0.546	0.216	0.320	0.280
Sex		0.830	0.404	0.074	0.958	0.056	0.112	0.282	0.247	0.004	0.472	0.002	0.018
GAA × Sex		0.634	0.547	0.314	0.936	0.806	0.758	0.249	0.698	0.434	0.815	0.848	0.101

Means followed by different letters in the same column are significantly different (*p* < 0.05). SEM = standard error of means, M = male, F = female.

**Table 4:** Impact of guanidinoacetic acid (GAA) on carcass characteristics (%) of male and female broiler chickens at 24 days of age.

GAA, %	Sex	Breast	Leg	Heart	Liver	Kidney	Pancreas	Gizzard	Proventriculus	Bursa of Fabricius	Thymus	Spleen
0	M	23.91	24.33	0.573	1.851	0.534	0.361	1.677	0.484	0.205	0.397	0.080
	F	24.93	25.16	0.542	2.304	0.660	0.347	1.708	0.498	0.193	0.466	0.096
0.06	M	23.61	24.64	0.633	1.921	0.597	0.316	1.763	0.514	0.222	0.378	0.088
	F	25.16	25.05	0.610	2.001	0.596	0.343	1.813	0.552	0.201	0.443	0.093
0.12	M	24.06	26.28	0.638	1.934	0.532	0.364	1.893	0.523	0.206	0.412	0.083
	F	24.23	24.51	0.541	1.958	0.534	0.356	1.651	0.497	0.183	0.424	0.080
SEM		0.465	0.201	0.012	0.540	0.017	0.006	0.036	0.010	0.007	0.013	0.004
Sex	M	23.86	25.08	0.615 ^a^	1.902	0.554	0.347	1.778	0.507	0.211	0.396	0.083
	F	24.77	24.91	0.564 ^b^	2.088	0.596	0.349	1.724	0.516	0.192	0.444	0.089
SEM		0.685	0.266	0.015	0.074	0.023	0.009	0.051	0.014	0.010	0.019	0.006
*p*-value												
GAA		0.969	0.321	0.065	0.534	0.196	0.124	0.512	0.215	0.584	0.808	0.665
Sex		0.351	0.643	0.023	0.082	0.198	0.895	0.462	0.665	0.174	0.077	0.462
GAA × Sex		0.840	0.056	0.311	0.203	0.200	0.375	0.188	0.401	0.927	0.631	0.641

Means followed by different letters in the same column are significantly different (*p* < 0.05). SEM= standard error of means, M = male, F = female.

**Table 5:** Impact of guanidinoacetic acid (GAA) on serum metabolites of male and female broiler chickens at 10 days of age.

GAA, %	Sex	Total Protein, g·dL^−1^	Albumin, g·dL^−1^	Globulin, g·dL^−1^	Glucose, mg·dL^−1^	Cholesterol, mg·dL^−1^	Uric Acid, mg·dL^−1^	Creatinine, mg·dL^−1^	T3, ng·mL^−1^	T4, µg·mL^−1^
0	M	2.531	1.234	1.298	289.0	186.8	3.799	0.346 ^ab^	7.136	22.78
	F	2.496	1.213	1.284	266.0	179.6	3.025	0.274 ^bc^	6.936	23.00
0.06	M	2.668	1.228	1.440	281.5	188.6	3.876	0.268 ^bc^	7.022	22.82
	F	2.548	1.339	1.209	292.8	177.5	3.366	0.362 ^a^	6.992	23.01
0.12	M	2.523	1.199	1.324	277.5	170.4	3.769	0.343 ^ab^	7.089	22.90
	F	2.401	1.471	0.930	278.0	171.8	3.181	0.299 ^abc^	6.934	22.99
SEM		0.043	0.029	0.046	4.425	3.683	0.155	0.011	0.027	0.026
Sex	M	2.574	1.220	1.354	282.7	181.9	3.815	0.319	7.082	22.83
	F	2.482	1.341	1.141	278.9	176.3	3.191	0.312	6.954	23.00
SEM		0.062	0.037	0.059	6.347	5.302	3.815	0.014	0.036	0.034
*p-*value										
GAA		0.394	0.237	0.129	0.613	0.326	0.853	0.898	0.883	0.556
Sex		0.298	0.058	0.054	0.681	0.457	0.051	0.712	0.066	0.061
GAA × Sex		0.899	0.088	0.185	0.292	0.786	0.939	0.003	0.380	0.528

Means followed by different letters in the same column are significantly different (*p* < 0.05). SEM= standard error of means, T3 = triiodothyronine, T4 = thyroxine, M = male, F = female.

**Table 6:** Impact of guanidinoacetic acid (GAA) on serum metabolites of male and female broiler chickens at 24 days of age.

GAA, %	Sex	Total Protein, g·dL^−1^	Albumin, g·dL^−1^	Globulin, g·dL^−1^	Glucose, mg·dL^−1^	Cholesterol, mg·dL^−1^	Uric Acid, mg·dL^−1^	Creatinine, mg·dL^−1^	T3, ng·mL^−1^	T4, µg·mL^−1^
0	M	2.505	1.363	1.142	226.5	165.1	2.355	0.315	6.650	23.10
	F	2.638	1.428	1.210	237.8	162.3	1.934	0.349	6.737	22.89
0.06	M	2.394	1.239	1.155	212.3	156.5	2.065	0.300	6.777	23.16
	F	2.658	1.363	1.295	235.8	176.5	2.616	0.397	6.482	22.97
0.12	M	2.450	1.309	1.141	195.6	169.9	3.000	0.365	6.563	23.10
	F	2.718	1.386	1.331	221.0	178.4	2.676	0.397	6.480	22.87
SEM		0.042	0.032	0.048	4.854	4.419	0.163	0.015	0.040	0.029
Sex	M	2.450	1.303	1.146	211.4	163.8	2.473	0.327	6.663	23.12
	F	2.671	1.392	1.279	231.5	172.4	2.409	0.381	6.566	22.91
SEM		0.057	0.046	0.070	6.540	6.392	0.230	0.021	0.053	0.035
*p*-value										
GAA		0.825	0.503	0.873	0.112	0.625	0.210	0.394	0.182	0.389
Sex		0.059	0.180	0.191	0.056	0.349	0.843	0.075	0.203	0.058
GAA × Sex		0.899	0.739	0.928	0.881	0.795	0.589	0.411	0.599	0.129

SEM = standard error of means, T3 = triiodothyronine, T4 = thyroxine, M = male, F = female.

**Table 7:** Impact of guanidinoacetic acid (GAA) on intestinal morphometry of male and female broiler chickens at 10 days of age.

GAA, %	Sex	VH, µm	VW, µm	CD, µm	VH/CD	SA, mm^2^	GC ^1^
0	M	404 ^d^	87.23 ^b^	51.88	8.010	0.114	106
	F	439 ^cd^	91.98 ^b^	74.51	6.389	0.129	117
0.06	M	517 ^a^	83.30 ^b^	54.97	9.635	0.136	128
	F	466 ^bc^	113 ^a^	72.44	6.958	0.166	130
0.12	M	505 ^ab^	94.81 ^ab^	59.02	9.360	0.150	125
	F	465 ^bc^	103 ^ab^	73.00	6.770	0.154	132
SEM		7.50	3.42	1.79	0.23	0.006	2.57
Sex							
Male		476 ^a^	103 ^a^	55.29 ^b^	9.002 ^a^	0.150 ^a^	126.5 ^a^
Female		457 ^b^	88.45 ^b^	73.32 ^a^	6.705 ^b^	0.133 ^b^	120.1 ^b^
SEM		4.35	1.98	1.03	0.13	0.003	1.49
GAA × Segment							
0 × Duodenum		475 ^abc^	82.24 ^bc^	53.14	9.237 ^b^	0.122	105 ^de^
0 × Jejunum		349 ^d^	61.63 ^d^	64.34	5.654 ^c^	0.069	100 ^e^
0 × Ileum		440 ^c^	124.9 ^a^	72.11	6.707 ^c^	0.174	129 ^bc^
0.06× Duodenum		498 ^a^	92.14 ^b^	54.51	9.306 ^b^	0.143	123 ^bc^
0.06 × Jejunum		495 ^ab^	67.16 ^cd^	60.84	8.623 ^b^	0.104	148 ^a^
0.06 × Ileum		482 ^abc^	136 ^a^	75.78	6.960 ^c^	0.206	118 ^cd^
0.12 × Duodenum		523 ^a^	94.59 ^b^	50.49	10.86 ^a^	0.155	121 ^c^
0.12 × Jejunum		448 ^abc^	80.22 ^bc^	73.51	6.572 ^c^	0.115	130 ^bc^
0.12 × Ileum		484 ^abc^	122	74.03	6.761 ^c^	0.187	136 ^ab^
SEM		7.77	2.37	1.76	0.20	0.005	2.80
Sex × Segment							
M × Duodenum		506	78.33 ^d^	46.35	11.15	0.125 ^c^	110
M × Jejunum		448	69.57 ^d^	56.96	8.070	0.101 ^d^	123
M × Ileum		472	117 ^b^	62.56	7.786	0.174 ^b^	126
F × Duodenum		491	101 ^c^	59.08	8.455	0.156 ^b^	122
F × Jejunum		414	69.77 ^d^	75.50	5.830	0.091 ^d^	128
F× Ileum		465	138 ^a^	85.38	5.832	0.203 ^a^	128
SEM		6.36	1.95	1.44	0.17	0.004	2.30
GAA × Sex × Segment							
0 × M × Duodenum		476	74.59 ^gh^	47.11 ^hi^	10.31 ^b^	0.111 ^efg^	97.16
0 × M × Jejunum		316	52.43 ^i^	52.35 ^ghi^	6.135 ^fgh^	0.052 ^h^	91.37
0 × M × Ileum		421	134 ^b^	56.18 ^fgh^	7.584 ^def^	0.180 ^bc^	129
0 × F × Duodenum		474	89.89 ^ef^	59.16 ^efgh^	8.163 ^de^	0.134 ^def^	112
0 × F × Jejunum		383	70.82 ^gh^	76.34 ^bcd^	5.173 ^h^	0.085 ^gh^	108
0 × F × Ileum		458	115.2 ^c^	88.04 ^ab^	5.830 ^gh^	0.169 ^bc^	129
0.06 × M × Duodenum		513	81.36 ^fg^	50.34 ^ghi^	10.21 ^b^	0.131 ^def^	118
0.06 × M × Jejunum		532	62.98 ^hi^	54.64 ^fghi^	10.06 ^bc^	0.105 ^fg^	150
0.06 × M × Ileum		507	105 ^cd^	59.93 ^efgh^	8.642 ^de^	0.170 ^bc^	117
0.06 × F × Duodenum		483	104 ^cde^	58.67 ^efgh^	8.407 ^de^	0.156 ^cd^	129
0.06 × F × Jejunum		459	71.35 ^gh^	67.04 ^def^	7.190 ^efg^	0.102 ^fg^	145
0.06 × F × Ileum		457	166 ^a^	91.62 ^a^	5.278 ^h^	0.241 ^a^	118
0.12 × M × Duodenum		530	79.04 ^fg^	41.60 ^i^	12.93 ^a^	0.132 ^def^	116
0.12 × M × Jejunum		497	93.30 ^def^	63.90 ^defg^	8.019 ^de^	0.145 ^cde^	129
0.12 × M × Ileum		488	112 ^c^	71.57 ^cde^	7.133 ^efg^	0.173 ^bc^	132
0.12 × F × Duodenum		515	110 ^c^	59.39 ^efgh^	8.794 ^cd^	0.178 ^bc^	125
0.12 × F × Jejunum		400	67.15 ^gh^	83.11 ^abc^	5.126 ^h^	0.085 ^gh^	130
0.12 × F × Ileum		480	132 ^b^	76.49 ^bcd^	6.389 ^fgh^	0.200 ^b^	139
SEM		11.00	3.38	2.47	0.29	0.007	3.99
*p*-value							
GAA		<0.001	0.009	0.25	<0.001	<0.001	<0.001
Sex		0.002	<0.001	<0.001	<0.001	0.001	0.003
GAA× Sex		>0.001	>0.001	0.214	0.106	0.132	0.508
GAA× Segment		>0.001	0.002	0.055	>0.001	0.168	>0.001
Sex× Segment		0.474	>0.001	0.125	0.397	0.003	0.438
GAA× Sex× Segment		0.064	>0.001	0.014	0.005	>0.001	0.694

Means followed by different letters in the same column are significantly different (*p* < 0.05). SEM = standard error of means, VH = villus height, VW = villus width, CD = crypt depth, SA = surface area, GC = goblet cells count, M = male, F = female. ^1^ GC = goblet cells number per villi from base towards the tip of the villi.

**Table 8:** Impact of guanidinoacetic acid (GAA) on intestinal morphometry of male and female broiler chickens at 24 days of age.

GAA,%	Sex	VH, µm	VW, µm	CD, µm	VH/CD	SA, mm^2^	GC ^1^
0	M	524 ^b^	85.65	56.17 ^b^	9.794 ^a^	0.143	126 ^ab^
	F	450 ^d^	73.64	49.04 ^c^	9.348 ^ab^	0.105	107 ^d^
0.06	M	576 ^a^	84.27	58.95 ^ab^	10.07 ^a^	0.153	131 ^a^
	F	455 ^d^	78.53	51.10 ^c^	9.024 ^b^	0.114	114 ^c^
0.12	M	561 ^a^	92.42	63.19 ^a^	9.582 ^ab^	0.164	124 ^b^
	F	484 ^c^	82.24	50.81 ^c^	9.975 ^a^	0.126	118 ^c^
SEM		6.13	3.05	0.98	0.23	0.005	1.84
Sex							
Male		554 ^a^	87.44 ^a^	50.31 ^b^	9.813	0.153 ^a^	127 ^a^
Female		463 ^b^	78.14 ^b^	59.44 ^a^	9.449	0.115 ^b^	113 ^b^
SEM		450	1.89	0.81	0.14	0.003	1.28
GAA × Segment							
0 × Duodenum		488 ^cde^	75.34	51.02 ^de^	9.631 ^c^	0.116	120 ^b^
0 × Jejunum		455 ^f^	68.37	49.20 ^e^	9.780 ^c^	0.099	122 ^b^
0 × Ileum		518 ^bc^	95.21	57.59 ^c^	9.301 ^c^	0.157	108 ^c^
0.06 × Duodenum		539 ^b^	80.51	51.23 ^de^	10.62 ^b^	0.137	130 ^a^
0.06 × Jejunum		465 ^ef^	69.62	49.46 ^e^	9.602 ^c^	0.104	126
0.06 × Ileum		542 ^b^	94.07	64.39 ^b^	8.413 ^d^	0.159	111 ^c^
0.12 × Duodenum		471 ^def^	89.32	43.13 ^f^	11.62 ^a^	0.133	128 ^a^
0.12 × Jejunum		502 ^cd^	73.93	53.76 ^cd^	9.527 ^c^	0.117	130 ^a^
0.12 × Ileum		593 ^a^	98.74	74.11 ^a^	8.193 ^d^	0.185	104 ^c^
Sex × Segment							
M × Duodenum		528 ^b^	88.00	51.82 ^c^	10.58	0.145	140 ^a^
M **×** Jejunum		515 ^b^	77.17	54.44 ^c^	9.937	0.125	135 ^a^
M × Ileum		619 ^a^	97.17	72.05 ^a^	8.927	0.190	105 ^d^
F × Duodenum		471 ^c^	75.45	45.10 ^d^	10.67	0.112	113 ^bc^
F **×** Jejunum		434 ^d^	64.12	47.17 ^d^	9.336	0.088	116 ^b^
F × Ileum		484 ^c^	94.84	58.67 ^b^	8.345	0.145	110 ^cd^
GAA × Sex × Segment							
0 × M × Duodenum		526 ^e^	81.01 ^defg^	55.87 ^def^	9.448	0.134 ^defgh^	136 ^ab^
0 × M × Jejunum		489 ^fg^	69.27 ^efgh^	51.84 ^fgh^	10.28	0.106 ^hijk^	133 ^ab^
0 × M × Ileum		558 ^cd^	106.7 ^a^	60.80 ^cd^	9.657	0.188 ^ab^	107 ^def^
0 × F × Duodenum		449 ^hi^	69.67 ^efgh^	46.18 ^i^	9.814	0.098 ^jk^	104 ^def^
0 × F × Jejunum		422 ^ij^	67.47 ^fgh^	46.57 ^i^	9.284	0.091 ^k^	109 ^cde^
0 × F × Ileum		479 ^fgh^	83.78 ^cdef^	54.38 ^ef^	8.944	0.126 ^efghi^	108 ^cdef^
0.06 × M × Duodenum		579 ^c^	84.96 ^bcde^	53.86 ^efg^	10.93	0.155 ^cde^	142 ^a^
0.06 × M × Jejunum		528 ^de^	80.81 ^defg^	52.91 ^fgh^	10.37	0.099 ^jk^	141 ^a^
0.06 × M × Ileum		621 ^b^	87.05 ^bcd^	70.09 ^b^	8.897	0.157 ^cd^	109 ^cde^
0.06 × F × Duodenum		498 ^ef^	76.07 ^defg^	48.60 ^hi^	10.31	0.120 ^fghij^	115 ^cd^
0.06 × F × Jejunum		402 ^j^	58.44 ^h^	46.01 ^i^	8.836	0.134 ^defgh^	111 ^cde^
0.06 × F × Ileum		463 ^gh^	101.1 ^ab^	58.68 ^cde^	7.930	0.171 ^bc^	113 ^cde^
0.12 × M × Duodenum		478 ^fgh^	98.03 ^abc^	45.73 ^i^	11.35	0.147 ^cdef^	141 ^a^
0.12 × M × Jejunum		527 ^e^	81.42 ^defg^	58.57 ^cde^	9.166	0.135 ^defg^	131 ^b^
0.12 × M × Ileum		678 ^a^	97.81 ^abc^	85.27 ^a^	8.441	0.211 ^a^	99.25 ^f^
0.12 × F × Duodenum		465 ^gh^	80.61 ^defg^	40.53 ^j^	11.88	0.118 ^ghijk^	115 ^cd^
0.12 × F × Jejunum		477 ^fgh^	66.45 ^gh^	48.94 ^ghi^	9.888	0.100 ^ijk^	128 ^b^
0.12 × F × Ileum		509 ^ef^	99.67 ^abc^	62.94 ^c^	8.226	0.160 ^cd^	109 ^cde^
SEM		10.48	5.211	1.717	0.393	0.009	3.207
*p*-value							
GAA		<0.001	0.03	0.012	0.52	0.001	0.03
Sex		<0.001	<0.001	<0.001	0.062	<0.001	<0.001
GAA × Sex		>0.001	0.566	0.014	0.007	0.989	0.001
GAA × Segment		>0.001	0.678	>0.001	>0.001	0.150	0.025
Sex × Segment		>0.001	0.135	0.001	0.221	0.469	>0.001
GAA × Sex × Segment		>0.001	0.003	0.001	0.602	0.020	0.038

Means followed by different letters in the same column are significantly different (*p* < 0.05). SEM = standard error of means, VH = villus height, VW = villus width, CD = crypt depth, SA = surface area, GC = goblet cells count, M = male, F = female. ^1^ GC = goblet cells number per villi from base towards the tip of the villi.

## Data Availability

The datasets that were generated for this study are available on request to the corresponding author.
